# Synthesis of Fe/Mg-Biochar Nanocomposites for Phosphate Removal

**DOI:** 10.3390/ma13040816

**Published:** 2020-02-11

**Authors:** Xuefeng Tao, Tao Huang, Bo Lv

**Affiliations:** 1 Faculty of Geosciences and Environmental Engineering, Southwest Jiaotong University, Chengdu 610031, China; TaoHuang365@outlook.com; 2 Chongqing Municipal Research Institute of Design, Chongqing 400020, China; lvbo1980@foxmail.com

**Keywords:** Fe/Mg-biochar nanocomposites, phosphate removal, biochar

## Abstract

Magnetic biochar derived from agricultural biomass has been recognized as a cost-effective biochar sorbent for phosphate removal. This study evaluated the use of novel Fe/Mg-biochar nanocomposites (WBC1x), prepared by impregnating ground walnut shell in a solution with a different molar ratio of Fe^2+^ to Mg^2+^, then pyrolyzing slowly, at a temperature of 600 °C, to remove phosphate. The results showed that MgO and Fe_3_O_4_ were loaded onto the biochar successfully through the impregnation-pyrolysis method and the composites were able to be separated easily by magnetic field. Meanwhile, a higher surface area and point of zero charge on WBC1x were observed compared to the non-magnetic biochar (WBC). Moreover, the isothermal adsorption and kinetics data further suggested the that phosphate adsorption onto WBC1x resulted from chemisorption. Additionally, the maximum phosphate adsorption capacity of WBC1x was 6.9 mg.g^−1^, obtained though the Langmuir–Freundlich model, which was threefold higher than WBC, where MgO addition could enhance the adsorption capacity of WBC1x markedly by improving the surface charge.

## 1. Introduction

The widespread occurrence of phosphate in the aquatic environment due to human activities may pose a threat to the ecosystem [1]. Chemical precipitation, biological consumption and physico-chemical adsorption methods have been developed to remove the phosphate in aqueous solution [2,3]. The application of chemical precipitation and biological consumption methods are constrained by factors such as substantial investment and maintaining cost, limited application condition, introduction of secondary pollutants [4] and low efficiency due to the less active living organism [5]. Therefore, the development of a sorption method to remove phosphate that is cost-effective and has a high efficiency, has therefore received increasing research focus.

The use of biochar as a cost-efficient adsorbent of organic pollutant has received increasing attention [6]. However, biochar in power form might not be a promising adsorbent for removing phosphate. For example, the use of a powered form of biochar released phosphate into the aqueous solution instead of removing it [7]. Furthermore, one drawback of using a powered form of biochar is separating the material after its application. Consequently, the development of magnetic biochar offers the possibility of addressing the difficulties faced by using biochar to remove phosphate. For example, in wastewater treatment studies where magnetic biochar was employed, the adsorbent and adsorbate could be easily separated [8,9]. Some studies have produced magnetic biochar via impregnation and low-temperature pyrolysis with ferrous chloride and ferric chloride, and magnesium chloride hexahydrate [5,10,11]; the magnetic biochars in some of these studies, comprised of nano-sized Fe_x_O_y_/MgO particles, exhibited a great affinity for phosphate in liquid solution [5,10], for example, magnetic biochar, synthesized under the temperature of 250 °C, removed 67.3% of phosphate, which is much higher than the removal rate of companion non-magnetic biochars (7.5%) [10]. Furthermore, as the point of zero charge (*pH_pzc_*) of MgO (approximately 12) is higher than Fe_3_O_4_ (around 7), the addition of a different ratio of Fe/Mg during magnetic biochar synthesis might modify the surface charge of magnetic biochar differently, thereby imposing different effects on the adsorption ability of magnetic biochar. A few studies have examined the effect of magnesium addition and pH on the efficiency of magnesium-decorated magnetic biochars in adsorbing phosphate [12]. However, factors such as the adsorbent amount and the presence of anions also have found to affect the sorption behavior of phosphate onto biochar.

Therefore, the aim of this study was to develop a magnetic biochar with the formation of nano-sized Fe_x_O_y_/MgO on the biochar surface. A walnut shell was chosen as the raw material of biochar because it is a widely available agricultural waste. Various techniques were used to characterize the physical–chemical properties of walnut shell biochar (WBC) and magnetic walnut shell biochar (WBC1x). Another focus of this study is to investigate the effect of the amount of WBC and WBC1x, Fe^2+^/Mg^2+^ ratio, pH and anionic salt on the adsorption of phosphate onto WBC and WBC1x.

## 2. Materials and Methods

### 2.1. Materials

FeCl_2_·4H_2_O and MgCl_2_·6H_2_O were purchased from Sinopharm Chemical Reagent Company (Chengdu, China). NaOH, HCl, KH_2_PO_4_ are analytical grade and were used as received. The N_2_ gas used during the synthesis of WBC/WBC1x was purchased from Hengyuan (Chengdu, China). All water mentioned in this work was 18.25 MΩ·cm^−1^ ultrapure water.

### 2.2. Synthesis of WBC and WBC1x

WBC was produced from air-dried and ground walnut shell through synthesis at 600 °C (50 °C·min^−1^ heating rate, 1 h residue time) inside a furnace (SK3-2-10-8, Zhuochi, Hangzhou, China) under an N_2_ environment (140 mL·min^−1)^. The biochar was ground and sieved in a size range of 60–100 μm. After being washed with 0.05 M HCl and NaOH, WBC was washed with water until the value of electrical conductivity was less than 5 μS·cm^−1^, followed by drying at 80 °C for 12 h.

To prepare a solution with three different mole ratios of Fe^2+^ to Mg^2+^, three 400 mL 0.04 M FeCl_2_·4H_2_O solutions were mixed with 0 M, 0.04 M, 0.08 M MgCl_2_·6H_2_O, respectively. An amount of 30 g air-dried and ground walnut shell was added the solution, followed by the adjustment of the pH of the mixture to 11.2 ± 0.03. After the mixture was kept at 30 °C for 6 h, WBC1x solid was harvested by filtration and dried at 80 °C for 12 h. The condition of WBC1x synthesis followed the method used for WBC synthesis, except the HCl and NaOH treatment. According to the mole ratios of Fe^2+^ to Mg^2+^ during WBC1x synthesis, WBC1x was expressed as WBC10, WBC11, and WBC12, respectively, where 10 means the mole ration of Fe^2+^ to Mg^2+^ is 1:0, 11 means the mole ration of Fe^2+^ to Mg^2+^ is 1:1, and 12 means the mole ration of Fe^2+^ to Mg^2+^ is 1:2.

### 2.3. WBC and WBC1x Characterization

The C, H, and N of washed WBC and WBC1x were analyzed using an element analyzer (Vario EL cube, Elementar, Hanau, Germany). The function groups of WBC and WBC1x were quantified using the Fourier transform infrared (FTIR) (NICOLET 6700, Thermo Fisher Scientific, Waltham, MA, USA). The specific surface areas, crystal structure and surface morphology of the adsorbents were determined and visualized using a surface area porosity analyzer (3H-2000PS2, Bei Shi De, Beijing, China), an X-ray diffraction analyzer (XRD) (Bruker D2 Phaser, Bruker AXE, Karlsruhe, Germany) and Scanning Electron Microscopy (SEM) (VEGA-3SBU, Tescan, Kohoutovice, Czech Republic), respectively. The synthesized WBC1x were separated by an external magnetic field. The point of zero charge (*pH_pzc_*) was determined using the method described by Liu et al. [13]: briefly, 0.05 g of WBC, WBC10, WBC11, and WBC20 were added to 25 mL 0.01 M NaNO_3_ in a 50 mL beaker, respectively, followed by the adjustment of the pH (pH_i_) to a range of 4–10 using 1 M NaOH and 1 M HNO_3_. The beakers were sealed with plastic wrap and were shaken at 120 rpm and 30 °C for 24 h. The final pH (pH_f_) of the solutions was measured by a pH Electrode (INESA, Shanghai, China) and pH_pzc_ could be determined at △pH = 0 from the graph of (pH_i_-pH_f_) against pH_i_. 

### 2.4. Batch Experiment

The stock solution of phosphate (100 mg/L) was prepared by dissolving K_2_HPO_4_ in water. All batch sorption experiments on phosphate were conducted in triplicate:

(1) To investigate the effect of adsorbent amounts on phosphate adsorption, 0.025, 0.050 and 0.100 g of WBC and WBC1x were added to 25 mL phosphate stock solution in a 50 mL beaker, respectively. The beakers were sealed in plastic wrap and shaken in a temperature-controlled gas bath at 120 rpm and 30 °C, and samples were taken at a preset time;
(2) To study the effect of pH, the pH of the KH_2_PO_4_ solution was adjusted in a range between 3 and 10 by using 1.00 mol/L NaOH or 1.00 mol/L HNO_3_ 0.05 g WBC1x was added to 25 mL adjusted phosphate solution in a 50 mL beaker, respectively. The beakers were sealed in plastic wrap and shaken in a temperature-controlled gas bath at 120 rpm and 30 °C, and samples were taken at preset time;
(3) To investigate the effect of anions (Cl^−^, SO_4_^2−^ and Cl^−^ + SO_4_^2−^), mixing a series of effective concentrations of anions (50, 100, 150, 200 and 250 mg/L) with 50 mg/L of KH_2_PO_4_. Then, 0.05 g WBC1x was added to 25 mL adjusted phosphate solution in a 50 mL beaker, respectively. The beakers were sealed in plastic wrap and shaken in a temperature-controlled gas bath at 120 rpm and 30 °C, and samples were taken at preset time;
(4) In the batch sorption experiments, 0.05 g WBC, WBC10, WBC11, and WBC20 were added to 25 mL phosphate stock solution in a 50 mL beaker, respectively. The beakers were sealed in plastic wrap and shaken in a temperature-controlled gas bath at 120 rpm and 30 °C, and samples were taken at preset time;
(5) n the isothermal adsorption experiments, 0.05 g of WBC, WBC10, WBC11, and WBC20 were added to 25 mL KH_2_PO_4_ solution with a concentration ranging from 5 to 400 mg·L^−1^, in a 50 mL beaker, respectively. The beakers were sealed in plastic wrap and shaken in a temperature-controlled gas bath at 120 rpm and 30 °C for 12 h, and samples were taken at preset time.

### 2.5. Regeneration of Spent WBC1x

An amount of 150 mL of KH_2_PO_4_ was added to 0.1 g WBC12. The mixture was sealed and mixed using an orbital mixer for 2 h at 120 rpm at a temperature of 30 °C. Then, the spent WBC12 was magnetically separated from the solution. The spent WBC12 was washed two times in ultrapure water, then dried at 80 °C for 24 h. The dried spent WBC12 was added to 200 mL of 0.5 mol/L NaOH, then sealed in the mixture and shaken for 2 h for the purpose of desorption. The concentration of phosphate before adsorption, after adsorption and after NaOH desorption was measured. The regenerated WBC12 was dried and regenerated 4 times following the same protocol described above. The calculation formulas for adsorption, desorption and desorption rate are as follows:

(1) $$q_{1}=\frac{c_{0}-c_{i}}{m}\times V;\left(i=1,2,3,4,5\right)$$

(2) $$q_{j}=\frac{c_{j}}{m}\times V;\left(j=1,2,3,4,5\right)$$

(3) $$\mathrm{Desorption}\;\mathrm{rate}=\frac{q_{j}}{q_{i}}\times 100\%$$

where *q_i_* is the adsorption amount of phosphorus at the *i*th time (mg/g), *q_j_* is the adsorption amount of phosphorus at the *j*th time (mg/g), *C*_0_ is the initial concentration of phosphorus (mg/L), *Ci* is the concentration of phosphorus after *i*th time adsorption (mg/L), *Cj* is the concentration of phosphorus after *j*th time desorption (mg/L), m is the mass of adsorbent (g), V is the volume of liquid (mL).

### 2.6. Analysis of Phosphate

All WBC samples were filtered through 0.45 μm Whatman (Whatman Inc., Piscataway, NJ, USA) and WBC1x samples were magnetically separated. The phosphate concentration in the filtered liquid sample was then analyzed by a spectrophotometer (UV-1100, Hengzheng, Changzhou, China) using the ascorbic acid method (ESSMethod 310.1 (USEPA, 1992)). The amount of phosphate adsorbed onto WBC/WBC1x was calculated based on the difference between the initial and final phosphate concentrations in the solution.

## 3. Results and Discussion

### 3.1. Physico-Chemical Properties of WBC1x

The physico-chemical properties of WBC and WBC1x were listed in Table 1. In WBC1x, O% is highest (11.66%) while C% is lowest in WBC1x; as a consequence, greater O/C is obtained in WBC1x compared to WBC. The higher O/C in WBC1x is consistent with the findings of [14] that the introduction of MgO and Fe_x_O_y_ contributed to the increase of O% in WBC1x. On the other hand, WBC1x has a higher surface area (ranging from 231.10 to 263.58 m^2^·g^−1^) compared to WBC (138.09 m^2^·g^−1^). The increase in surface area was due to the modification of the pore structure when synthesizing WBC1x in the presence of FeCl_2_·4H_2_O under alkaline conditions (pH > 11) [15,16].

As shown in Table 1, WBC has a *pH_pzc_* of 4.8, implying that it is acidic, which is correlated closely with the raw material, temperature and time during the synthesis of WBC. However, all WBC1x are alkaline, since the value of *pH_pzc_* is greater than eight. This phenomenon might be attributed to the introduction of a high zero charge point (FeCl_2_·4H_2_O and MgCl_2_·6H_2_O) which changed the acid base of WBC1x, or the fact that the type of surface charge on WBC1x altered in a solution with a different pH. The *pH_pzc_* of WBC1x increased with the increase in Mg addition in the synthesis process, which is because the *pH_pzc_* of MgO (approximately 12) [17] is greater than Fe_3_O_4_ (around seven) [18], indicating that the *pH_pzc_* of WBC1x was positively related to the amount of MgCl_2_·6H_2_O addition.

**Figure 1:**
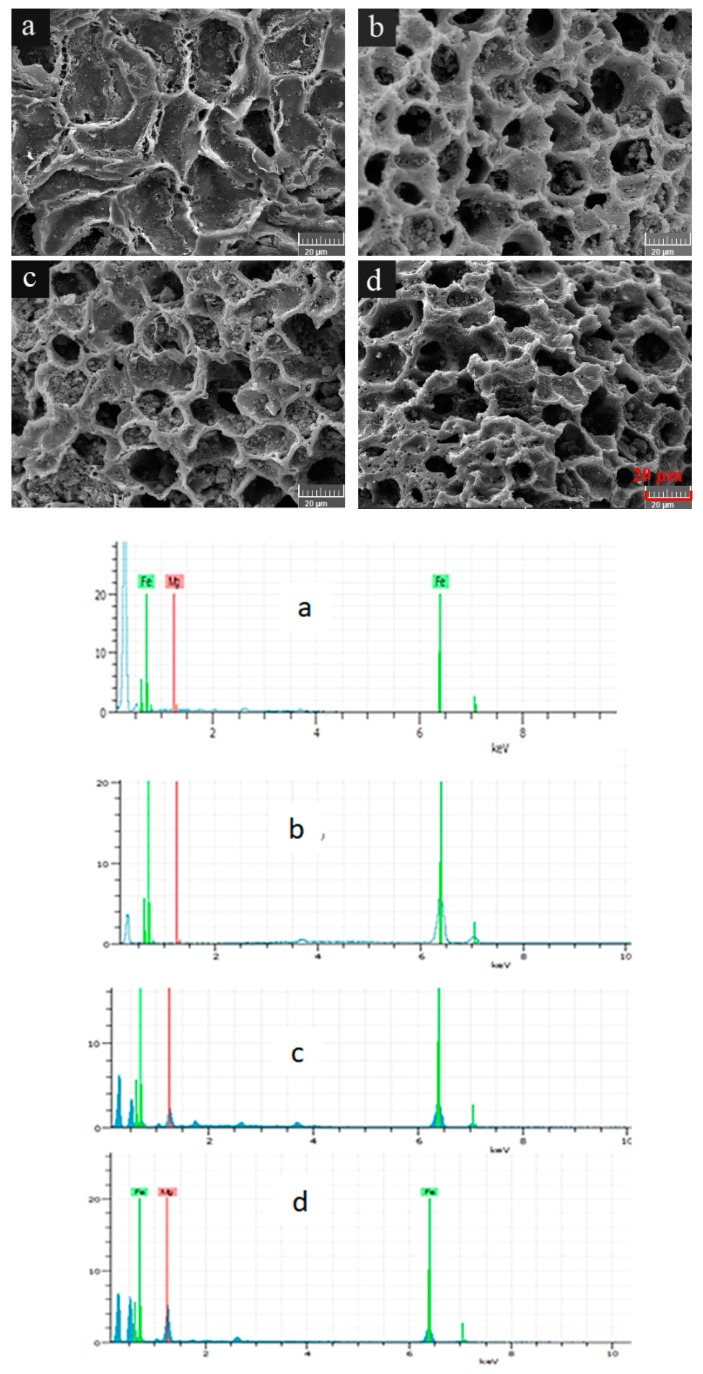
Morphological structures of WBC and WBC1x. (**a**) WBC, (**b**) WBC10, (**c**) WBC11 and (**d**) WBC12.

To compare the morphological structures of WBC and WBC1x, their surfaces were visualized (Figure 1). WBC had smooth, shallow, concave surfaces which were scatted with a certain number of small microholes (Figure 1), while the surface morphologies of WBC1x demonstrated a honeycomb-like structure (Figure 1b–d). The honeycomb-like structure had well developed pores containing rough surfaces and a great number of pore volumes, which could provide active sites for the adsorption of phosphate.

**Figure 2:**
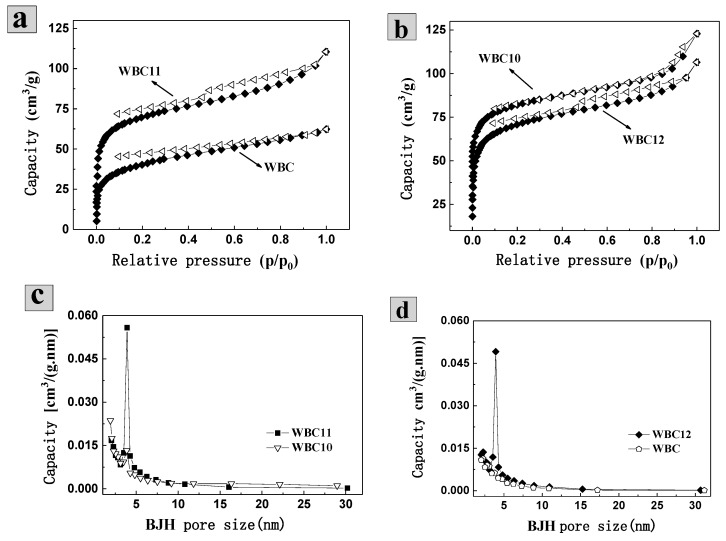
N_2_-adsorption/desorption isotherms and aperture profile of WBC and WBC1x. (**a**) N_2_-adsorption/desorption isotherms of WBC (♦) and WBC 11(Δ); (**b**) N_2_-adsorption/desorption isotherms of WBC10(Δ) and WBC 12 (♦); (**c**) BHJ aperture distribution of WBC1 0(∎) and WBC 11(▽); (**d**) BHJ aperture distribution of WBC (⬠) and WBC 12 (♦).

The isothermal adsorption of N_2_ found that the N_2_ adsorption capacity of WBC is 62.30 mL·g^−1^, which was lower than N_2_ adsorption capacity of WBC10, WBC11 and WBC12 (122.87, 110.46 and 106.52 mL·g^−1^, respectively) (Figure 2a,b). In addition, the isotherm of WBC and WBC1x were type-IV according to the International Union of Pure and Applied Chemistry (UPAC) classification, indicating that all the materials had a strong force with nitrogen gas and most of them had a mesoporous structure. All materials demonstrated a certain extent of H4 type hysteresis ring (around P/P_0_ = 0.5), indicating that the pores of the adsorbent are slits in some layered structures. The appearance of the hysteresis ring is due to the condensation effect of capillary-triggered N_2_ molecules condensing below the normal pressure and filling the mesoporous channels. The combination of N_2_-adsorption isotherms in the aperture profile (Figure 2c,d) of these materials further revealed that WBC and WBC1x are mesoporous in general, which agreed with the surface area and morphological structures of WBC and WBC1x discussed previously.

**Figure 3:**
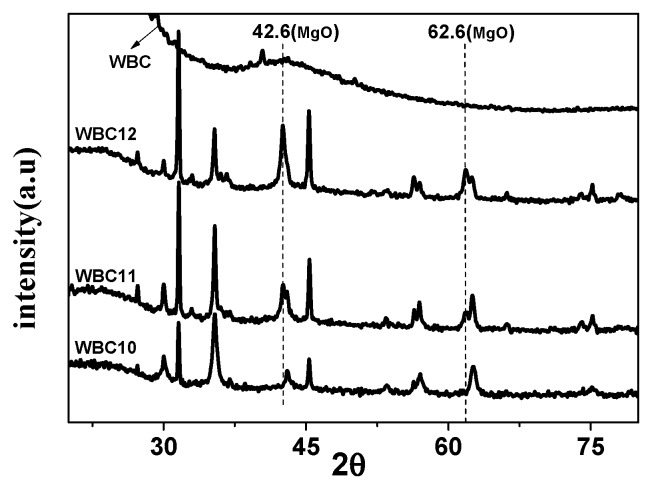
XRD patterns of WBC and WBC1x.

**Figure 4:**
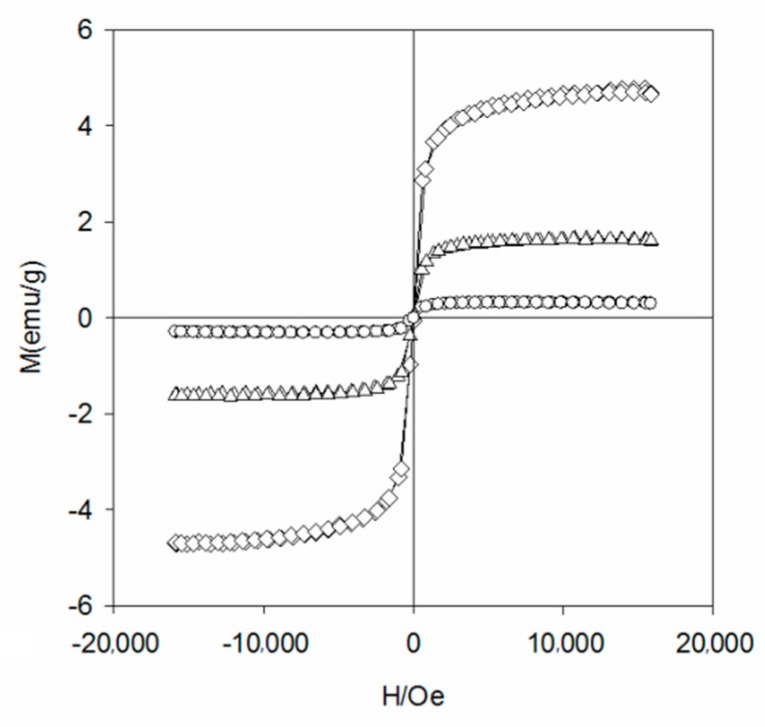
The magnetization curve of WBC10(◊), WBC11(Δ) and WBC12(○).

**Figure 5:**
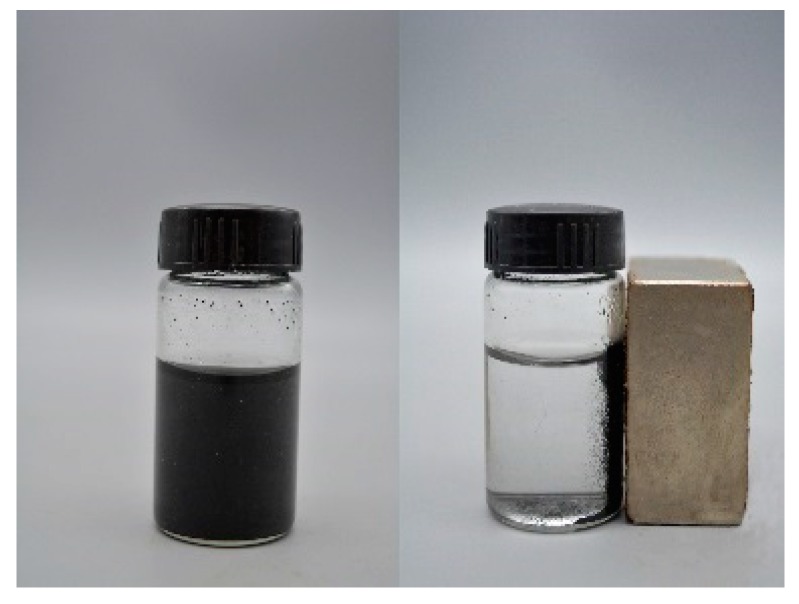
Magnetic separation of WBC12 after adsorbing phosphate from liquid solution.

To study the crystal structure of WBC and WBC1x, their XRD patterns were shown in Figure 3 and it can be observed that the diffraction pattern of WBC did not show any crystalline peaks, indicating its amorphous nature. Sharp and strong peaks of magnetic Fe_3_O_4_ (2θ = 30.1, 31.6, 45.4, 56.3, 57, 75.3) were detected on WBC1x, suggesting that the Fe_3_O_4_ is highly crystalline. Signals of MgO were also detected at 42.5° and 62.6° for WBC11 and WBC12, as their intensity rose with the increase in MgCl_2_·6H_2_O addition. Additionally, a peak of γ-Fe_2_O_3_ (θ = 35.5) [8] was also found on WBC1x. Since the presence of non-magnetic γ-Fe_2_O_3_ could reduce the magnetic saturation of the magnetic adsorbent, its magnetic property was measured in external magnetic fields. There was no obvious hysteresis ring in the magnetization curve, all of them were S-shaped, and the remanence and coercivity were close to zero, indicating excellent superparamagnetic ability (Figure 4). The saturation magnetizations of WBC10, WBC11, WBC12 were 4.78, 1.65, 0.32 emg/g, respectively, indicating that the higher Mg^2+^ addition resulted in a weaker magnetic separation. It also implies that the use of a magnet or the application of an external magnetic field could recover WBC1x after treatment [19]. Figure 5 visualizes the magnetic separation of WBC12 from the liquid solution, implying the possibility of separating and reusing WBC12 after the sorption treatment, which held true for WBC10 and WBC11. 

**Figure 6:**
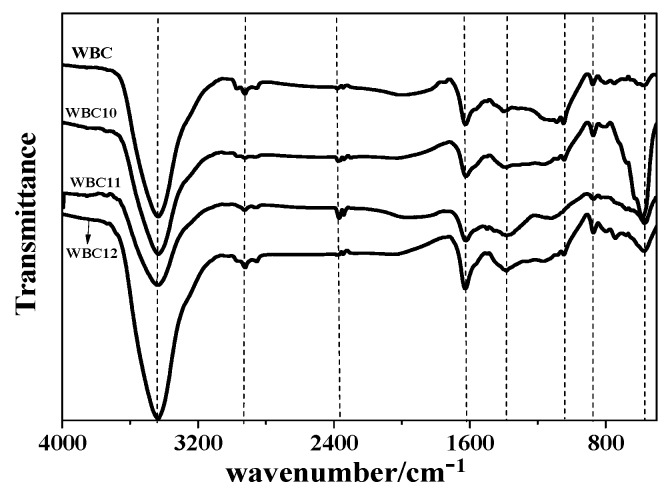
Fourier transform infrared (TIR) spectra of WBC and WBC1x.

To obtain detailed characteristics of WBC and WBC1x, their FTIR spectra are illustrated in Figure 6. A wide and sharp peak was detected at 3400 cm^−1^ for WBC and WBC1x, indicating the existence of ^–^OH function groups or alcohols and aldehydes [20]. The adsorption peak on WBC1x at 570 cm^−1^ was assigned as a stretching vibration of Fe-O [21], while no peak was observed at 570 cm^−1^ for WBC (Figure 6). Various bands in the spectra represent the vibrations of functional groups in the biochars [20]: –COOH or –CH_2_ (1390 cm^−1^), –CH_2_ (2850 cm^−1^), –CH_3_ (2926 cm^−1^), –COOH and C=O (2300–2400 cm^−1^), aromatic C=C/C=O (1623 cm^−1^) [22], C-O–C or alcohols, ethers, esters (1100–1000 cm^−1^), and furan (875 cm^−1^). Among the three types of WBC1x, the greatest peak intensity was observed for WBC10, revealing the reduction in Fe_x_O_y_ load on WBC11 and WBC12. The lower peak intensity in the spectra of WBC11 and WBC12 is possibly because of the reduction in Fe-O peak intensity, due to the competition of MgO and Fe_x_O_y_ on the WBC1x surface.

### 3.2. Effect of Adsorbent Amount, Fe^2+^/Mg^2+^ Ratio, pH and Anions on Phosphate Adsorption

**Figure 7:**
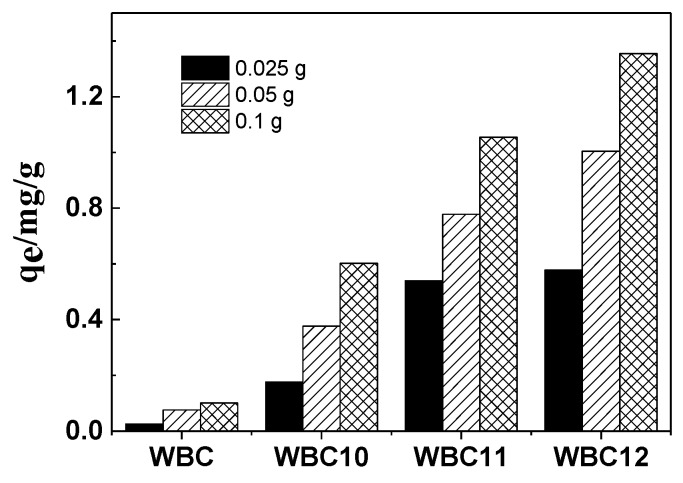
Effects of different adsorbent doses on phosphate adsorption.

The effect of three doses (0.025, 0.050 and 0.100 g) of biochar and magnetic biochar on phosphate adsorption is shown in Figure 7. The sorption of phosphate onto WBC, WBC10, WBC11 and WBC12 grew by 4.0, 3.4, 1.9 and 2.3 folds when the adsorbent addition increased from 0.025 to 0.100 g, which was mainly due to an increase in the quantity of WBC and WBC1x in the liquid solution. Compared to WBC, a greater increase in phosphate adsorption was obtained for WBC1x, which might be attributed to the reduction in adsorption competition and mass transfer, which was due to the rise in the positive charge resulting from the higher zero charge point of WBC1x (Table 1). Additionally, the greatest increase came in WBC, which may be because the adsorption of WBC was more affected by mass transfer resistance; more specifically, the increase in the adsorption dose reduced the mass transfer resistance, so as to promote the adsorption of phosphate. In Figure 7, we can also observe that the rise in Fe^2+^/Mg^2+^ ratio also increased the phosphate adsorption, which might be attributed to the addition of Mg^2+^, which occupied the adsorption position of the biochar.

**Figure 8:**
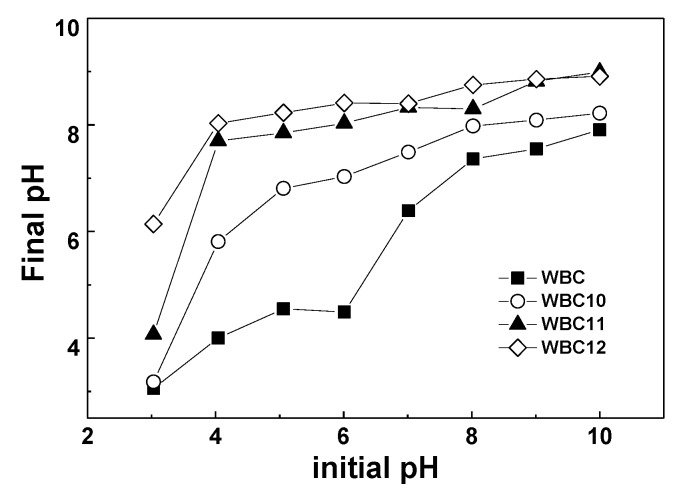
The effect of adsorption capacity in different pH (and pH change after adsorption).

The effect of pH on the adsorption capacity of WBC and WBC1x is shown in Figure 8. The phosphate adsorption decreased with the rise in the pH of the solution from 3–10. In particular, for WBC11 and WBC12, phosphate adsorption decreased dramatically when the pH rose from 3 to 4, which might be attributed to the presence of a form of phosphate in solution, the acid base of the biochar surface functional group and the zero charge point of biochar. When the pH was lower than the *pH_PZC_*, the adsorbent surface was positive, and vice versa. Since the *pH_pzc_* of MgO and Fe_3_O_4_ is approximately 12 [17] and 7 [18], respectively, the surface charge capacity of biochar loaded with these two metal oxides is improved, which provides an opportunity for the electrostatic adsorption of negatively charged anions [5]. When the pH ranges between 3 and 10, phosphate is mainly in the form of H_2_PO_4_^−^, HPO_4_^2−^, and PO_4_^3−^, which is conducive to the adsorption of phosphate onto biochar. However, since the total number of adsorption sites is constant, the number of OH^−^ in the solution increases continuously with the rise in pH, thus resulting in the adsorption competition between OH^−^ and phosphate. Moreover, when the pH is beyond *pH_PZC_*, the surface of the adsorbent is negatively charged, which reduces the adsorption of phosphate, resulting in a decrease in the adsorption capacity. Therefore, pH could affect phosphate adsorption by influencing the nature of the biochar and the existence of a form of phosphate.

**Figure 9:**
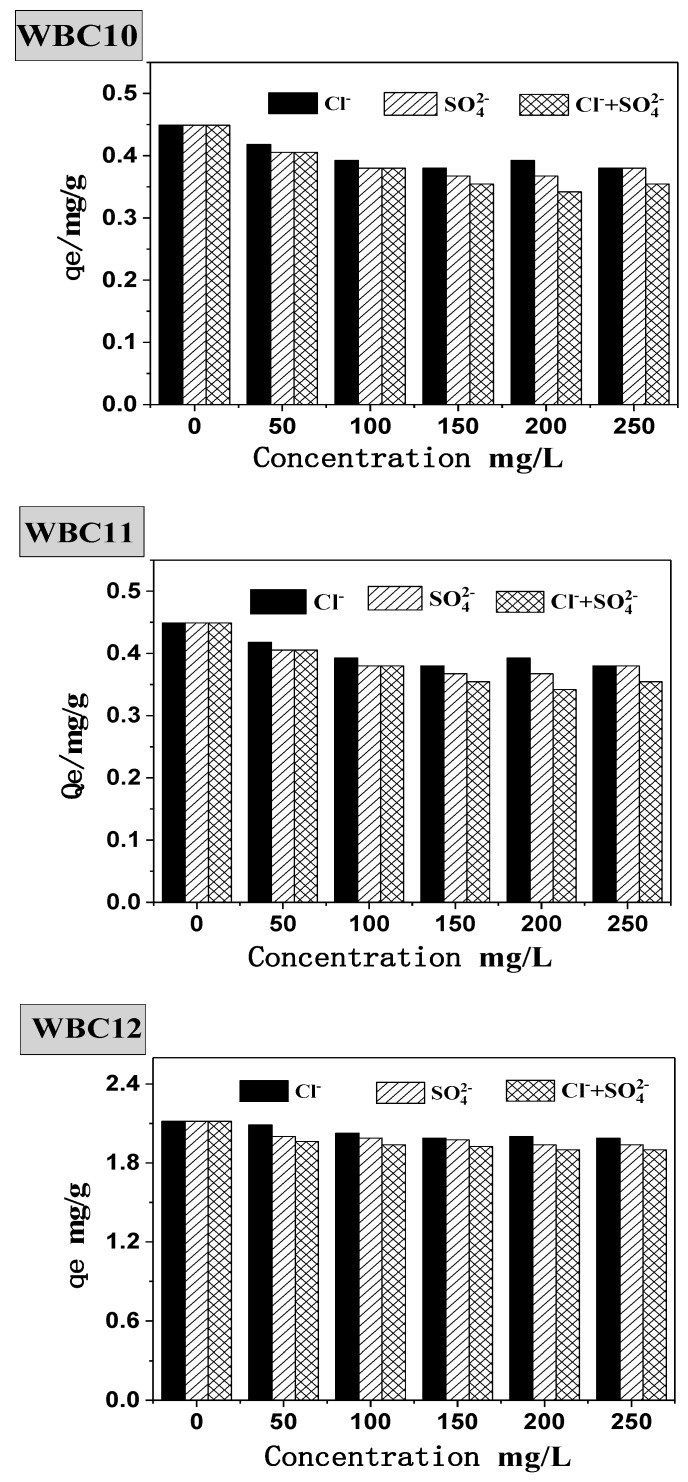
Effect of coexisting anions on phosphate adsorption.

Natural bodies of water often contain various anions, which will undoubtedly interfere with the adsorption behavior of phosphate onto the adsorbent. Therefore, this paper studied the presence of Cl^−^, SO_4_^2−^ and Cl^−^ + SO_4_^2−^ during the adsorption of phosphate onto WBC1x, as shown in Figure 9. Without the presence of Cl^−^, SO_4_^2−^, the equilibrium adsorption capacity of WBC10, WBC11 and WBC12 was 0.45, 0.75 and 2.12 mg/g, respectively. The addition of Cl^−^, SO_4_^2−^ and Cl^−^ + SO_4_^2−^ was attributed to a decrease in the equilibrium adsorption capacity of WBC1x, for example, the equilibrium adsorption capacity of WBC10, WBC11 and WBC12 decreased to 0.38, 0.68 and 1.99 mg/g, respectively, with the addition of Cl^−^. The influence of anions on the equilibrium adsorption capacity of WBC1x was not significant, and the influence anions was in the order of Cl^−^ > SO_4_^2−^> Cl^−^ + SO_4_^2−^. From the perspective of WBC1x, the materials that tend to be affected by the anion salt the most are in the following order: WBC10, WBC11, WBC12. The reduction in the equilibrium adsorption capacity in the presence of anions was due to the adsorption completion between anions and phosphate in the solution. Meanwhile, the possible reason that anions imposed the minimum impact on WBC12 adsorbent is because WBC12, synthesized with the highest magnesium addition (0.08 M MgCl_2_·6H_2_O), had the greatest surface charge capacity due to its highest *pH_PZC_* value (9.10) among WBC1x.

### 3.3. Adsorption Kinetics

**Figure 10:**
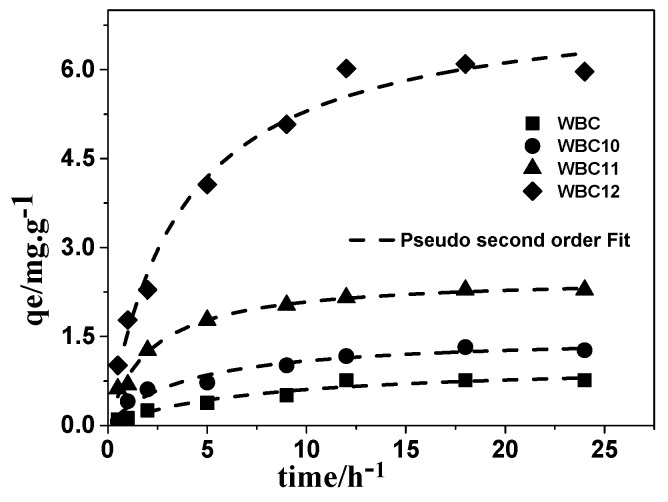
Adsorption kinetic and modeling for phosphate onto WBC and WBC1x.

To understand the adsorption mechanism, the amount of phosphate adsorbed per gram of adsorbent—after adsorption onto WBC and WBC1x—as a function of time was plotted, and then fitted through three mathematics models, namely pseudo-first order, pseudo-second order, and infra-particle diffusion. The kinetic parameters of phosphate adsorption on WBC and WBC1x are given in Table 2. Among the kinetic models used, the pseudo-second order model described the experimental data best (Figure 10), with an *R*^2^ value no less than 0.952, revealing that chemisorption is the rate controlling step in the adsorption of phosphate onto WBC and WBC1x. The pseudo-first order and intra-particle diffusion model also described the experiment data reasonably well, with *R*^2^ values greater than 0.859, indicating that parameters such as pore structures and pore volume, etc., also play a certain role in the adsorption capacity of WBC and WBC1x. As shown in the literature, the surface charge and function groups of the adsorbent might affect the adsorption of phosphate. Since the WBC1x had a *pHpzc* greater than 8.0 after introducing a different mole ratio of Fe^2+^/Mg^2+^, a colloidal interface with a positive charge would be produced after the formation of surface hydroxyl compounds in the presence of the KH_2_PO_4_ solution (pH < 8), thereby enhancing the adsorption of phosphate onto WBC1x. No significant difference was observed between the function groups of WSB and WBC1x. Consequently, our results were consistent with the findings of Yao et al. [5] that the adsorption ability of both WBC and WBC1x is more likely related to their surface charge. 

### 3.4. Adsorption Isotherm

The equilibrium concentration data obtained from the adsorption of phosphate onto WBC and WBC1x were fitted through three isotherm models, namely Langmuir, Freundlich and Langmuir–Freundlich [5,23], and these equations are shown as follows:

(4) $$q_{e}=\frac{q_{\max}K_{L}C_{e}}{1+K_{L}C_{e}}$$

(5) $$q_{e}=K_{F}C_{e}^{1/n}$$

(6) $$q_{e}=\frac{q_{\max}KC_{e}^{1/n}}{1+KC_{e}^{1/n}}$$

where *q_e_* is equilibrium adsorption capacity(mg/g), *q_max_* is the maximum adsorption capacity (mg/g), *C_e_* is the equilibrium concentration of adsorbate (mg/L), *K_L_* is the Langmuir isotherm constant (L/mg), *K_F_* is the Freundlich isotherm constant (mg/g(L/mg)^1/*n*^), *K* is the Langmuir–Freundlich isotherm constant (1/(L/mg)^1/*n*^) and *1/n* is the measure of adsorption intensity.

**Figure 11:**
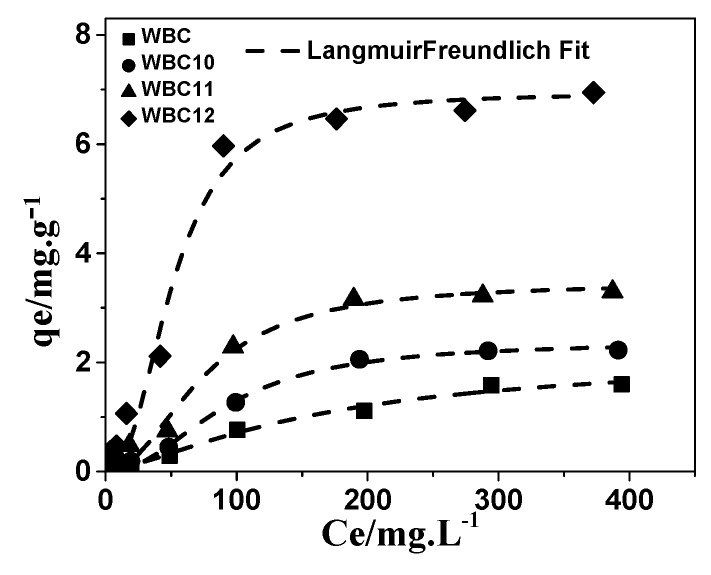
Adsorption isotherms of phosphate onto WBC and WBC1x.

Among the three models, the Langmuir–Freundlich model has the best description of the adsorption of phosphate onto WBC and WBC1x, with an *R*^2^ value greater than 0.972, as shown in Table 3 and Figure 11. Based on the Freundlich parameters, the value of *1/n* declined with the increase in Mg^2+^ addition, revealing that the increase in the number of adsorption sites in WBC1x, or the adsorption intensity of phosphate, would promote the adsorption process [24]. However, the value of *K_F_* increased with the increase in Mg^2+^ addition, which was in line with the rise in the MgO peak intensity after increasing the amount of MgCl_2_·6H_2_O addition, as described in Section 3.1. This result indicates that the amount of Mg^2+^ addition was the key factor that determined the adsorption capacity of WBC1x.

### 3.5. Regeneration of Spent WBC1x

**Figure 12:**
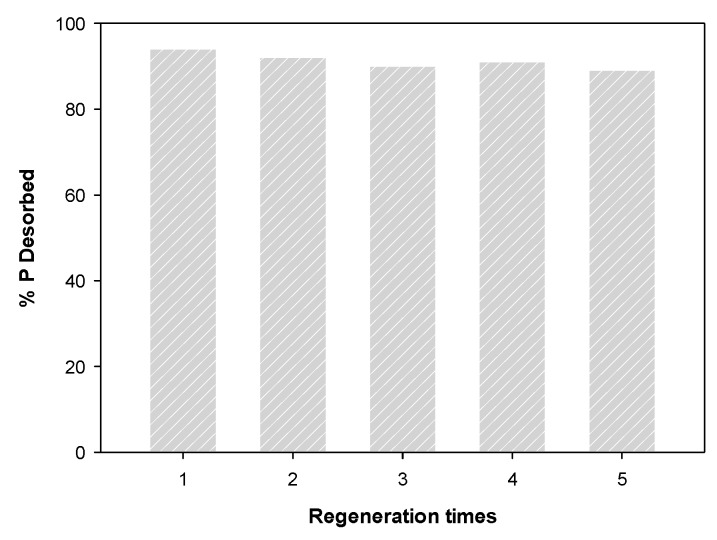
Regeneration of WBC12.

The result of the regeneration of phosphate-saturated WBC12 using NaOH are shown in Figure 12. The desorption rate was over 89% after five desorption experiments, indicating that WBC12 has high regeneration capacity and can be recycled, which corresponds to the physical adsorption rate obtained in the isothermal adsorption experiment. 

## 4. Conclusions

This study successfully synthesized a novel magnetic adsorbent (WBC1x) by using agricultural waste (a walnut shell) and Fe/Mg nanocomposites to compare their ability in phosphate uptake. The following conclusions can be drawn:

(1) WBC1x was prepared through a combination of impregnation and slow pyrolysis at a temperature of 600 °C, which successfully loaded MgO and Fe_3_O_4_ onto WBC. As a consequence, all WBC1x have magnetic separation characteristics;
(2) The introduction of Fe^2+^ and Mg^2+^ greatly increased the specific surface area and *pH_pzc_* of WBC1x, wherein the value of *pH_pzc_* positively correlated with the amount of Mg^2+^ addition;
(3) Phosphate sorption onto WBC and WBC1x was mainly determined by chemisorption. The sorption of phosphate onto WBC1x, which was greater than onto WBC, was highly Mg^2+^ addition-dependent, wherein a higher Mg^2+^ addition was more favorable than a low Mg^2+^ addition.

## Figures and Tables

**Table 1:** Physico-chemical properties of WBC and WBC1x.

Adsorbent	C%	H%	O%	N%	H/C	O/C	BET Surface Area (m^2^.g^−1^)	Average Pore Diameter (nm)	*pH_PZC_*
WBC	84.23	2.96	11.66	0.15	0.04	0.14	138.09	2.79	4.80
WBC10	76.59	2.77	12.73	0.14	0.04	0.17	263.58	2.88	8.00
WBC11	76.50	2.74	13.10	0.13	0.04	0.17	229.71	2.98	8.90
WBC12	51.79	3.09	20.73	0.06	0.06	0.40	231.10	2.85	9.10

**Table 2:** Kinetic parameters for pseudo-first order, pseudo-second order, and intra-particle diffusion mechanism of phosphate onto WBC and WBC1x.

Adsorbent	Pseudo-First Order			Pseudo-Second Order			Intra-Particle Diffusion		
	*q_e_* (mg.g^−1)^	*K*_1_ (h^−1^)	*R* ^2^	*q_e_* (mg.g^−1)^	*K*_2_ (g.mg^−1^.h^−1^)	*R* ^2^	*K* (g.mg^−1^.h^−1/2^)	*C*	*R* ^2^
WBC	0.809 ± 0.002	0.146 ± 0.003	0.954	1.041 ± 0.001	0.134 ± 0.005	0.952	0.178 ± 0.001	−0.010 ± 0.001	0.924
WBC10	1.265 ± 0.005	0.224 ± 0.001	0.933	1.511 ± 0.002	0.170 ± 0.001	0.954	0.278 ± 0.005	0.093 ± 0.006	0.897
WBC11	2.196 ± 0.001	0.403 ± 0.003	0.966	2.512 ± 0.005	0.193 ± 0.001	0.987	0.422 ± 0.002	0.534 ± 0.005	0.859
WBC12	6.050 ± 0.010	0.246 ± 0.002	0.979	7.217 ± 0.008	0.038 ± 0.002	0.981	1.288 ± 0.009	0.661 ± 0.002	0.893

**Table 3:** Adsorption isotherm parameters of phosphate to WBC and WBC1x.

Isotherm Model	Langmuir			Freundlich			Langmuir-Freundlich		
Parameters	*q_max_* (mg.g^−1^)	*K_L_*	*R* ^2^	*K_F_* (L.mg^−1^)	*1/n*	*R* ^2^	*q_max_* (mg.g^−1^)	*K* (L.mg^−1^)	*R* ^2^
WBC	3.198 ± 0.002	0.003 ± 0.001	0.984	0.025 ± 0.001	0.712 ± 0.002	0.967	2.136 ± 0.010	8.400 ± 0.011	0.988
WBC10	3.661 ± 0.006	0.005 ± 0.001	0.965	0.061 ± 0.002	0.624 ± 0.002	0.922	2.376 ± 0.06	5.799 ± 0.007	0.995
WBC11	4.725 ± 0.003	0.008 ± 0.001	0.957	0.154 ± 0.001	0.535 ± 0.001	0.904	3.464 ± 0.005	1.227 ± 0.009	0.975
WBC12	8.871 ± 0.011	0.013 ± 0.002	0.943	0.489 ± 0.001	0.469 ± 0.001	0.862	6.945 ± 0.012	8.996 ± 0.013	0.972
